# Bis[1,2-bis­(eth­oxy­carbon­yl)ethene-1,2-dithiol­ato-κ^2^ *S*,*S*′]bis­(η^5^-penta­methyl­cyclo­penta­dien­yl)tetra-μ_3_-sulfido-diiron(IV)diiron(III)(3 *Fe*—*Fe*)

**DOI:** 10.1107/S1600536813005564

**Published:** 2013-03-02

**Authors:** Shohei Ito, Nozomu Hisamichi, Tsugiko Takase, Shinji Inomata

**Affiliations:** a Faculty of Symbiotic Systems Science, Fukushima University, 1 Kanayagawa, Fukushima 960-1296, Japan; b Center for Practical and Project-Based Learning, Cluster of Science and Technology, Fukushima University, 1 Kanayagawa, Fukushima 960-1296, Japan

## Abstract

The title compound, [Fe_4_(C_10_H_15_)_2_(C_8_H_10_O_4_S_2_)_2_S_4_], contains a twisted Fe_4_S_4_ cubane-like core. A twofold rotation axis passes through the Fe_4_S_4_ core, completing the coordination of the four Fe atoms with two penta­methyl­cyclo­penta­dienyl ligands and two chelating dithiol­ate ligands. There are three short Fe—Fe and three long Fe⋯Fe contacts in the Fe_4_S_4_ core, suggesting bonding and non-bonding inter­actions, respectively. The Fe—S bonds in the Fe_4_S_4_ core range from 2.1523 (5) to 2.2667 (6) Å and are somewhat longer than the Fe—S bonds involving the dithiol­ate ligand.

## Related literature

For details of the synthesis, see: Inomata *et al.* (1995 ▶). For related structures, see: Inomata *et al.* (1990 ▶, 1994 ▶). For general background to compounds with iron–sulfur cubane-type clusters, see: Holm (1977 ▶); Holm *et al.* (1990 ▶).
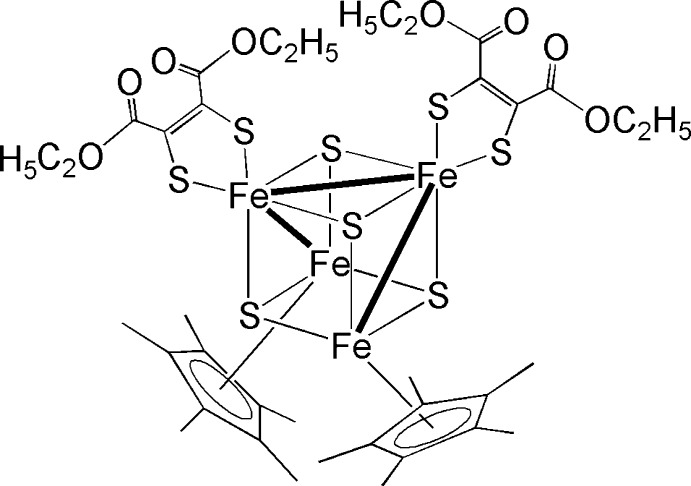


## Experimental

### 

#### Crystal data

[Fe_4_(C_10_H_15_)_2_(C_8_H_10_O_4_S_2_)_2_S_4_]
*M* *_r_* = 1090.65
Monoclinic, 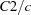

*a* = 23.4532 (5) Å
*b* = 10.4466 (2) Å
*c* = 18.3113 (3) Å
β = 90.6186 (7)°
*V* = 4486.14 (15) Å^3^
*Z* = 4
Mo *K*α radiation
μ = 1.69 mm^−1^
*T* = 296 K
0.30 × 0.20 × 0.20 mm

#### Data collection

Rigaku R-AXIS RAPID diffractometer
Absorption correction: multi-scan (*REQAB*; Jacobson, 1998 ▶) *T*_min_ = 0.560, *T*_max_ = 0.714
21429 measured reflections
5111 independent reflections
4694 reflections with *I* > 2σ(*I*)
*R*_int_ = 0.044

#### Refinement

*R*[*F* ^2^ > 2σ(*F* ^2^)] = 0.030
*wR*(*F* ^2^) = 0.080
*S* = 1.06
5111 reflections
254 parameters
H-atom parameters constrained
Δρ_max_ = 0.50 e Å^−3^
Δρ_min_ = −0.33 e Å^−3^

### 

Data collection: *RAPID-AUTO* (Rigaku, 2006 ▶); cell refinement: *RAPID-AUTO*; data reduction: *RAPID-AUTO*; program(s) used to solve structure: *SIR97* (Altomare *et al.*, 1999 ▶); program(s) used to refine structure: *SHELXL97* (Sheldrick, 2008 ▶); molecular graphics: *ORTEP-3 for Windows* (Farrugia, 2012 ▶); software used to prepare material for publication: *CrystalStructure* (Rigaku, 2006 ▶).

## Supplementary Material

Crystal structure: contains datablock(s) global, I. DOI: 10.1107/S1600536813005564/wm2725sup1.cif

Structure factors: contains datablock(s) I. DOI: 10.1107/S1600536813005564/wm2725Isup2.hkl

Additional supplementary materials:  crystallographic information; 3D view; checkCIF report

## Figures and Tables

**Table 1:** Selected bond lengths (Å)

Fe1—Fe1^i^	3.3743 (3)
Fe1—Fe2	2.7253 (4)
Fe1—Fe2^i^	3.2683 (3)
Fe1—S1	2.1956 (5)
Fe1—S1^i^	2.2551 (5)
Fe1—S2	2.1749 (5)
Fe2—Fe2^i^	2.7619 (3)
Fe2—S1	2.2736 (5)
Fe2—S2	2.1523 (5)
Fe2—S2^i^	2.2667 (6)
Fe2—S3	2.1541 (5)
Fe2—S4	2.1934 (6)

Symmetry code: (i) 
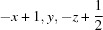
.

**Fig. 1.**
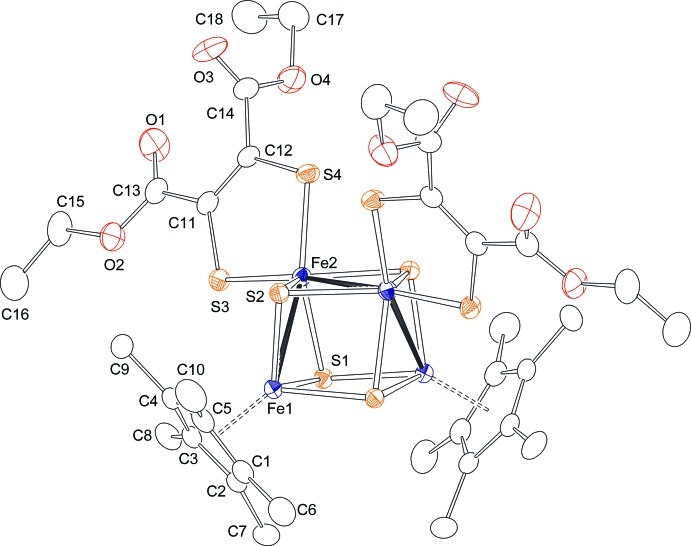
The molecular structure of the title compound, with atom labels and displacement ellipsoids at the 30% probability level. All hydrogen atoms were omitted for clarity. Solid lines indicate short Fe—Fe contacts.

## supplementary crystallographic information

### Comment

Iron-sulfur cubane-type clusters have extensively been investigated as model systems of metal-containing proteins (Holm, 1977; Holm *et al.*, 1990). Among these compounds, the Fe_4_S_4_ core is usually surrounded by the same supporting ligand (*L*) yielding a moiety Fe_4_S_4_*L*_4_. However, mixed-ligand-type clusters are rather rare. Previously, we succeeded to prepare this type of iron-sulfur cluster from the reaction of (C_5_Me_5_)_2_Fe_2_(CO)_4_ ((Cp*)_2_Fe_2_(CO)_4_) with S_8_ and diphenylacetylene (Inomata *et al.*, 1990; 1994). One of the products was [Fe_4_(Cp*)_2_(Ph_2_C_2_S_2_)_2_(µ_3_-S)_4_], in which two Cp* ligands and two diphenyldithiolate ligands are additionally bonded to the Fe_4_S_4_ core. In order to expand our research on this subject, we prepared a cluster containg bis(ethoxycarbonyl)dithiolate ligands instead of diphenyldithiolate ligands. Here we report the structural details of the title compound [Fe_4_(C_10_H_15_)_2_(C_8_H_10_O_4_S_2_)_2_S_4_] or [Fe_4_(Cp*)_2_{(EtO_2_C)_2_C_2_S_2_)}_2_(µ_3_-S)_4_], (I).

Compound (I) contains a twisted Fe_4_S_4_ cubane-like core surrounded by two Cp* ligands and two dithiolato ligands {(EtO_2_C)_2_C_2_S_2_} (Fig. 1). A crystallographic twofold rotation axis passes through the Fe_4_S_4_ core and completes the coordination environment of all iron atoms. There are three iron—iron bonds of 2.7253 (4) and 2.7619 (3) Å (Table 1). The remaining three Fe···Fe distances are very long (3.2683 (3) and 3.3743 (3) Å), indicating no bonding interactions (Table 1). The iron—sulfur distances in the Fe_4_S_4_ core range from 2.1523 (5) to 2.2667 (6) Å and are normal values (Table 1). On the other hand, the distances between iron and sulfur in the dithiolato ligand are somewhat short (2.1541 (5) and 2.1934 (6) Å) (Table 1).

### Experimental

The title cluster compound was prepared according to the literature method (Inomata *et al.*, 1995) by using diethyl acetylenedicarboxylate instead of dimethyl acetylenedicarboxylate.

### Refinement

All hydrogen atoms were placed in calculated positions with C—H distances of 0.96 Å for H atoms on methyl groups and 0.97 Å for those on methylene groups. The *U*_iso_(H) values were fixed at 1.2 times the *U*_eq_(C) values of the carbon atoms to which they are covalently bonded.

### Crystal data

**Table:**

[Fe_4_(C_10_H_15_)_2_(C_8_H_10_O_4_S_2_)_2_S_4_]	*F*(000) = 2248.00
*M**_r_* = 1090.65	*D*_x_ = 1.615 Mg m^−^^3^
Monoclinic, *C*2/*c*	Mo *K*α radiation, λ = 0.71075 Å
Hall symbol: -C 2yc	Cell parameters from 18942 reflections
*a* = 23.4532 (5) Å	θ = 3.1–27.5°
*b* = 10.4466 (2) Å	µ = 1.69 mm^−^^1^
*c* = 18.3113 (3) Å	*T* = 296 K
β = 90.6186 (7)°	Block, black
*V* = 4486.14 (15) Å^3^	0.30 × 0.20 × 0.20 mm
*Z* = 4	

### Data collection

**Table:**

Rigaku R-AXIS RAPID diffractometer	4694 reflections with *I* > 2σ(*I*)
Detector resolution: 10.00 pixels mm^-1^	*R*_int_ = 0.044
ω scans	θ_max_ = 27.5°
Absorption correction: multi-scan (*REQAB*; Jacobson, 1998)	*h* = −30→30
*T*_min_ = 0.560, *T*_max_ = 0.714	*k* = −13→13
21429 measured reflections	*l* = −23→23
5111 independent reflections	

### Refinement

**Table:**

Refinement on *F*^2^	H-atom parameters constrained
*R*[*F*^2^ > 2σ(*F*^2^)] = 0.030	*w* = 1/[σ^2^(*F*_o_^2^) + (0.037*P*)^2^ + 5.9377*P*] where *P* = (*F*_o_^2^ + 2*F*_c_^2^)/3
*wR*(*F*^2^) = 0.080	(Δ/σ)_max_ = 0.002
*S* = 1.06	Δρ_max_ = 0.50 e Å^−^^3^
5111 reflections	Δρ_min_ = −0.33 e Å^−^^3^
254 parameters	

### Special details

**Table:**

Refinement. Refinement was performed using all reflections. The weighted *R*-factor (*wR*) and goodness of fit (*S*) are based on *F*^2^. *R*-factor (gt) are based on *F*. The threshold expression of *F*^2^ > 2.0 σ(*F*^2^) is used only for calculating *R*-factor (gt).

### Fractional atomic coordinates and isotropic or equivalent isotropic displacement parameters (Å^2^)

**Table:**

	*x*	*y*	*z*	*U*_iso_*/*U*_eq_	
Fe1	0.512641 (12)	0.27575 (3)	0.159470 (14)	0.02745 (7)	
Fe2	0.449295 (12)	0.07723 (3)	0.210955 (14)	0.02769 (7)	
S1	0.43972 (2)	0.29022 (4)	0.23321 (3)	0.02894 (11)	
S2	0.53399 (2)	0.07354 (4)	0.16691 (3)	0.03038 (11)	
S3	0.38310 (2)	0.09718 (5)	0.12928 (3)	0.03767 (13)	
S4	0.42061 (2)	−0.11994 (5)	0.22914 (3)	0.03867 (13)	
O1	0.25875 (9)	−0.1373 (2)	0.08613 (13)	0.0745 (6)	
O2	0.29459 (8)	0.02895 (19)	0.02550 (11)	0.0590 (4)	
O3	0.33671 (9)	−0.35409 (19)	0.13925 (13)	0.0668 (5)	
O4	0.29995 (10)	−0.2666 (2)	0.23969 (11)	0.0675 (5)	
C1	0.56426 (10)	0.4192 (2)	0.10976 (12)	0.0416 (5)	
C2	0.50644 (10)	0.4620 (2)	0.11373 (11)	0.0382 (4)	
C3	0.47177 (10)	0.3774 (2)	0.07124 (11)	0.0388 (4)	
C4	0.50767 (12)	0.2819 (2)	0.04178 (11)	0.0414 (5)	
C5	0.56502 (11)	0.3076 (2)	0.06575 (12)	0.0442 (5)	
C6	0.61503 (13)	0.4854 (3)	0.14245 (16)	0.0642 (7)	
C7	0.48533 (15)	0.5815 (2)	0.14991 (15)	0.0594 (7)	
C8	0.40936 (12)	0.3974 (2)	0.05670 (16)	0.0555 (6)	
C9	0.48970 (16)	0.1760 (2)	−0.00894 (14)	0.0658 (8)	
C10	0.61697 (14)	0.2362 (3)	0.04239 (17)	0.0709 (9)	
C11	0.34615 (9)	−0.0459 (2)	0.12687 (12)	0.0364 (4)	
C12	0.36250 (9)	−0.1409 (2)	0.17332 (12)	0.0351 (4)	
C13	0.29508 (10)	−0.0580 (2)	0.07860 (13)	0.0425 (5)	
C14	0.33153 (10)	−0.2667 (2)	0.18025 (14)	0.0430 (5)	
C15	0.24678 (14)	0.0240 (3)	−0.02537 (17)	0.0716 (8)	
C16	0.2579 (2)	0.1231 (4)	−0.0829 (2)	0.0961 (13)	
C17	0.26445 (15)	−0.3807 (3)	0.25262 (19)	0.0764 (9)	
C18	0.20954 (15)	−0.3640 (3)	0.2128 (2)	0.0809 (9)	
H1	0.6029	0.5612	0.1675	0.077*	
H2	0.6410	0.5084	0.1044	0.077*	
H3	0.6338	0.4290	0.1764	0.077*	
H4	0.5163	0.6223	0.1754	0.071*	
H5	0.4702	0.6387	0.1136	0.071*	
H6	0.4560	0.5599	0.1839	0.071*	
H7	0.3950	0.3287	0.0269	0.067*	
H8	0.4038	0.4772	0.0317	0.067*	
H9	0.3894	0.3990	0.1022	0.067*	
H10	0.5225	0.1265	−0.0222	0.079*	
H11	0.4725	0.2119	−0.0521	0.079*	
H12	0.4627	0.1218	0.0151	0.079*	
H13	0.6502	0.2752	0.0638	0.085*	
H14	0.6197	0.2389	−0.0099	0.085*	
H15	0.6144	0.1488	0.0582	0.085*	
H16	0.2115	0.0425	−0.0004	0.086*	
H17	0.2439	−0.0604	−0.0473	0.086*	
H18	0.2415	0.2033	−0.0683	0.115*	
H19	0.2409	0.0963	−0.1284	0.115*	
H20	0.2982	0.1331	−0.0889	0.115*	
H21	0.2576	−0.3905	0.3045	0.092*	
H22	0.2838	−0.4567	0.2353	0.092*	
H23	0.1826	−0.3224	0.2440	0.097*	
H24	0.1950	−0.4463	0.1985	0.097*	
H25	0.2155	−0.3126	0.1701	0.097*	

### Atomic displacement parameters (Å^2^)

**Table:**

	*U*^11^	*U*^22^	*U*^33^	*U*^12^	*U*^13^	*U*^23^
Fe1	0.03035 (15)	0.02725 (14)	0.02472 (14)	0.00053 (10)	−0.00037 (11)	0.00188 (10)
Fe2	0.02636 (14)	0.02784 (15)	0.02878 (14)	−0.00189 (10)	−0.00376 (10)	−0.00136 (10)
S1	0.0284 (2)	0.0298 (2)	0.0286 (2)	0.00303 (17)	−0.00230 (18)	−0.00054 (17)
S2	0.0330 (2)	0.0297 (2)	0.0284 (2)	0.00387 (18)	0.00056 (18)	−0.00021 (17)
S3	0.0369 (2)	0.0381 (2)	0.0377 (2)	−0.0058 (2)	−0.0122 (2)	0.0029 (2)
S4	0.0398 (2)	0.0289 (2)	0.0470 (2)	−0.0048 (2)	−0.0122 (2)	0.0007 (2)
O1	0.0465 (10)	0.0993 (16)	0.0771 (13)	−0.0314 (11)	−0.0221 (9)	0.0159 (12)
O2	0.0545 (10)	0.0647 (11)	0.0571 (10)	−0.0097 (9)	−0.0274 (8)	0.0042 (9)
O3	0.0607 (12)	0.0478 (10)	0.0923 (15)	−0.0169 (9)	0.0138 (10)	−0.0262 (10)
O4	0.0772 (14)	0.0671 (12)	0.0584 (11)	−0.0350 (11)	0.0157 (10)	−0.0072 (9)
C1	0.0466 (12)	0.0448 (12)	0.0332 (10)	−0.0098 (9)	0.0023 (9)	0.0112 (8)
C2	0.0525 (12)	0.0319 (9)	0.0299 (9)	−0.0015 (9)	−0.0011 (8)	0.0076 (7)
C3	0.0481 (12)	0.0378 (10)	0.0305 (9)	−0.0012 (9)	−0.0051 (8)	0.0094 (8)
C4	0.0636 (14)	0.0360 (10)	0.0247 (9)	−0.0021 (9)	0.0008 (9)	0.0052 (7)
C5	0.0504 (13)	0.0483 (12)	0.0341 (10)	0.0047 (10)	0.0125 (9)	0.0121 (9)
C6	0.0592 (16)	0.0761 (19)	0.0573 (15)	−0.0296 (15)	−0.0040 (13)	0.0159 (14)
C7	0.092 (2)	0.0336 (12)	0.0527 (14)	0.0044 (12)	0.0071 (14)	0.0020 (10)
C8	0.0519 (14)	0.0576 (15)	0.0567 (14)	0.0024 (12)	−0.0162 (12)	0.0211 (12)
C9	0.116 (2)	0.0464 (14)	0.0354 (12)	−0.0124 (15)	0.0032 (14)	−0.0037 (10)
C10	0.072 (2)	0.080 (2)	0.0621 (17)	0.0207 (16)	0.0324 (15)	0.0133 (15)
C11	0.0296 (9)	0.0423 (11)	0.0371 (10)	−0.0047 (8)	−0.0029 (8)	−0.0075 (8)
C12	0.0305 (9)	0.0353 (10)	0.0394 (10)	−0.0050 (8)	−0.0013 (8)	−0.0080 (8)
C13	0.0338 (11)	0.0514 (12)	0.0420 (11)	−0.0028 (9)	−0.0055 (9)	−0.0085 (9)
C14	0.0380 (11)	0.0391 (11)	0.0518 (12)	−0.0085 (9)	−0.0034 (9)	−0.0045 (9)
C15	0.0638 (18)	0.093 (2)	0.0575 (16)	0.0007 (16)	−0.0333 (14)	−0.0024 (16)
C16	0.123 (3)	0.090 (2)	0.074 (2)	0.005 (2)	−0.046 (2)	0.006 (2)
C17	0.076 (2)	0.081 (2)	0.0722 (19)	−0.0367 (18)	0.0011 (17)	0.0134 (17)
C18	0.0600 (19)	0.093 (2)	0.090 (2)	−0.0129 (18)	0.0156 (17)	0.012 (2)

### Geometric parameters (Å, º)

**Table:**

Fe1—Fe1^i^	3.3743 (3)	C4—C9	1.502 (3)
Fe1—Fe2	2.7253 (4)	C5—C10	1.495 (4)
Fe1—Fe2^i^	3.2683 (3)	C11—C12	1.360 (3)
Fe1—S1	2.1956 (5)	C11—C13	1.486 (3)
Fe1—S1^i^	2.2551 (5)	C12—C14	1.508 (3)
Fe1—S2	2.1749 (5)	C15—C16	1.503 (5)
Fe1—C1	2.136 (2)	C17—C18	1.483 (5)
Fe1—C2	2.122 (2)	C6—H1	0.960
Fe1—C3	2.150 (2)	C6—H2	0.960
Fe1—C4	2.158 (2)	C6—H3	0.960
Fe1—C5	2.147 (2)	C7—H4	0.960
Fe2—Fe2^i^	2.7619 (3)	C7—H6	0.960
Fe2—S1	2.2736 (5)	C7—H5	0.960
Fe2—S2	2.1523 (5)	C8—H7	0.960
Fe2—S2^i^	2.2667 (6)	C8—H8	0.960
Fe2—S3	2.1541 (5)	C8—H9	0.960
Fe2—S4	2.1934 (6)	C9—H10	0.960
S3—C11	1.728 (2)	C9—H11	0.960
S4—C12	1.709 (2)	C9—H12	0.960
O1—C13	1.198 (3)	C10—H13	0.960
O2—C13	1.330 (3)	C10—H14	0.960
O2—C15	1.451 (3)	C10—H15	0.960
O3—C14	1.189 (3)	C15—H16	0.970
O4—C14	1.323 (3)	C15—H17	0.970
O4—C17	1.474 (4)	C16—H18	0.960
C1—C2	1.431 (3)	C16—H19	0.960
C1—C5	1.417 (3)	C16—H20	0.960
C1—C6	1.496 (3)	C17—H21	0.970
C2—C3	1.426 (3)	C17—H22	0.970
C2—C7	1.500 (3)	C18—H23	0.960
C3—C4	1.416 (3)	C18—H24	0.960
C3—C8	1.500 (3)	C18—H25	0.960
C4—C5	1.435 (3)		
			
Fe1···Fe1^i^	3.3743 (3)	Fe1···Fe2	2.7253 (4)
Fe2···Fe2^i^	2.7619 (3)	Fe1···Fe2^i^	3.2683 (3)
Fe1^i^···Fe2^i^	2.7253 (4)	Fe1^i^···Fe2	3.2683 (3)
			
Fe2—Fe1—S1	53.736 (13)	C2—C3—C4	107.8 (2)
Fe2—Fe1—S1^i^	90.938 (15)	C2—C3—C8	124.1 (2)
Fe2—Fe1—S2	50.595 (15)	C4—C3—C8	127.9 (2)
Fe2—Fe1—C1	174.13 (6)	Fe1—C4—C3	70.52 (11)
Fe2—Fe1—C2	143.01 (6)	Fe1—C4—C5	70.12 (12)
Fe2—Fe1—C3	113.29 (6)	Fe1—C4—C9	127.37 (16)
Fe2—Fe1—C4	110.15 (6)	C3—C4—C5	108.14 (19)
Fe2—Fe1—C5	135.48 (6)	C3—C4—C9	126.2 (2)
S1—Fe1—S1^i^	80.87 (2)	C5—C4—C9	125.6 (2)
S1—Fe1—S2	102.04 (2)	Fe1—C5—C1	70.25 (13)
S1—Fe1—C1	131.50 (6)	Fe1—C5—C4	70.93 (13)
S1—Fe1—C2	97.42 (6)	Fe1—C5—C10	128.82 (18)
S1—Fe1—C3	94.83 (6)	C1—C5—C4	108.0 (2)
S1—Fe1—C4	125.31 (7)	C1—C5—C10	126.0 (2)
S1—Fe1—C5	159.56 (6)	C4—C5—C10	125.7 (2)
S1^i^—Fe1—S2	84.20 (2)	S3—C11—C12	118.52 (16)
S1^i^—Fe1—C1	92.67 (6)	S3—C11—C13	119.31 (16)
S1^i^—Fe1—C2	108.28 (5)	C12—C11—C13	122.0 (2)
S1^i^—Fe1—C3	146.55 (6)	S4—C12—C11	119.90 (16)
S1^i^—Fe1—C4	152.77 (7)	S4—C12—C14	116.30 (16)
S1^i^—Fe1—C5	113.81 (6)	C11—C12—C14	123.80 (19)
S2—Fe1—C1	125.22 (6)	O1—C13—O2	123.8 (2)
S2—Fe1—C2	158.40 (6)	O1—C13—C11	124.1 (2)
S2—Fe1—C3	128.84 (6)	O2—C13—C11	112.2 (2)
S2—Fe1—C4	95.83 (6)	O3—C14—O4	125.6 (2)
S2—Fe1—C5	93.85 (6)	O3—C14—C12	124.3 (2)
C1—Fe1—C2	39.26 (8)	O4—C14—C12	110.0 (2)
C1—Fe1—C3	65.39 (8)	O2—C15—C16	106.7 (2)
C1—Fe1—C4	65.05 (8)	O4—C17—C18	108.4 (2)
C1—Fe1—C5	38.65 (8)	C1—C6—H1	109.5
C2—Fe1—C3	38.99 (8)	C1—C6—H2	109.5
C2—Fe1—C4	64.89 (8)	C1—C6—H3	109.5
C2—Fe1—C5	65.17 (8)	H1—C6—H2	109.5
C3—Fe1—C4	38.37 (8)	H1—C6—H3	109.5
C3—Fe1—C5	64.99 (8)	H2—C6—H3	109.5
C4—Fe1—C5	38.95 (9)	C2—C7—H4	109.5
Fe1—Fe2—Fe2^i^	73.111 (11)	C2—C7—H6	109.5
Fe1—Fe2—S1	51.138 (15)	C2—C7—H5	109.5
Fe1—Fe2—S2	51.334 (14)	H4—C7—H6	109.5
Fe1—Fe2—S2^i^	105.375 (16)	H4—C7—H5	109.5
Fe1—Fe2—S3	94.456 (17)	H6—C7—H5	109.5
Fe1—Fe2—S4	159.452 (19)	C3—C8—H7	109.5
Fe2^i^—Fe2—S1	89.620 (16)	C3—C8—H8	109.5
Fe2^i^—Fe2—S2	53.196 (16)	C3—C8—H9	109.5
Fe2^i^—Fe2—S2^i^	49.487 (14)	H7—C8—H8	109.5
Fe2^i^—Fe2—S3	165.837 (19)	H7—C8—H9	109.5
Fe2^i^—Fe2—S4	100.674 (17)	H8—C8—H9	109.5
S1—Fe2—S2	100.26 (2)	C4—C9—H10	109.5
S1—Fe2—S2^i^	81.730 (19)	C4—C9—H11	109.5
S1—Fe2—S3	87.59 (2)	C4—C9—H12	109.5
S1—Fe2—S4	149.40 (2)	H10—C9—H11	109.5
S2—Fe2—S2^i^	102.65 (2)	H10—C9—H12	109.5
S2—Fe2—S3	113.75 (2)	H11—C9—H12	109.5
S2—Fe2—S4	109.02 (2)	C5—C10—H13	109.5
S2^i^—Fe2—S3	143.35 (2)	C5—C10—H14	109.5
S2^i^—Fe2—S4	83.36 (2)	C5—C10—H15	109.5
S3—Fe2—S4	88.64 (2)	H13—C10—H14	109.5
Fe1—S1—Fe1^i^	98.59 (2)	H13—C10—H15	109.5
Fe1—S1—Fe2	75.126 (18)	H14—C10—H15	109.5
Fe1^i^—S1—Fe2	92.386 (19)	O2—C15—H16	110.4
Fe1—S2—Fe2	78.071 (18)	O2—C15—H17	110.4
Fe1—S2—Fe2^i^	94.73 (2)	C16—C15—H16	110.4
Fe2—S2—Fe2^i^	77.32 (2)	C16—C15—H17	110.4
Fe2—S3—C11	106.95 (7)	H16—C15—H17	108.6
Fe2—S4—C12	105.87 (7)	C15—C16—H18	109.5
C13—O2—C15	116.4 (2)	C15—C16—H19	109.5
C14—O4—C17	117.0 (2)	C15—C16—H20	109.5
Fe1—C1—C2	69.86 (12)	H18—C16—H19	109.5
Fe1—C1—C5	71.10 (13)	H18—C16—H20	109.5
Fe1—C1—C6	127.30 (17)	H19—C16—H20	109.5
C2—C1—C5	107.7 (2)	O4—C17—H21	110.0
C2—C1—C6	125.9 (2)	O4—C17—H22	110.0
C5—C1—C6	126.4 (2)	C18—C17—H21	110.0
Fe1—C2—C1	70.88 (12)	C18—C17—H22	110.0
Fe1—C2—C3	71.55 (12)	H21—C17—H22	108.4
Fe1—C2—C7	127.60 (15)	C17—C18—H23	109.5
C1—C2—C3	108.28 (18)	C17—C18—H24	109.5
C1—C2—C7	126.9 (2)	C17—C18—H25	109.5
C3—C2—C7	124.6 (2)	H23—C18—H24	109.5
Fe1—C3—C2	69.46 (11)	H23—C18—H25	109.5
Fe1—C3—C4	71.12 (12)	H24—C18—H25	109.5
Fe1—C3—C8	128.94 (16)		

Symmetry code: (i) −*x*+1, *y*, −*z*+1/2.

## References

[bb1] Altomare, A., Burla, M. C., Camalli, M., Cascarano, G. L., Giacovazzo, C., Guagliardi, A., Moliterni, A. G. G., Polidori, G. & Spagna, R. (1999). *J. Appl. Cryst.* **32**, 115–119.

[bb2] Farrugia, L. J. (2012). *J. Appl. Cryst.* **45**, 849–854.

[bb3] Holm, R. H. (1977). *Acc. Chem. Res.* **12**, 427–434.

[bb4] Holm, R. H., Ciurli, S. & Weigel, J. A. (1990). *Prog. Inorg. Chem.* **38**, 1–74.

[bb5] Inomata, S., Hiyama, K., Tobita, H. & Ogino, H. (1994). *Inorg. Chem.* **33**, 5337–5342.

[bb6] Inomata, S., Takano, H., Hiyama, K., Tobita, H. & Ogino, H. (1995). *Organometallics*, **14**, 2112–2114.

[bb7] Inomata, S., Tobita, H. & Ogino, H. (1990). *J. Am. Chem. Soc.* **112**, 6145–6146.

[bb8] Jacobson, R. (1998). *REQAB* Private communication to the Rigaku Corporation, Tokyo, Japan.

[bb9] Rigaku (2006). *CrystalStructure* and *RAPID-AUTO* Rigaku Corporation, Tokyo, Japan.

[bb10] Sheldrick, G. M. (2008). *Acta Cryst.* A**64**, 112–122. 10.1107/S0108767307043930 18156677

